# Chengai Nomogram: A Comprehensive Study of Uroflow Parameters and Characteristics in Healthy Adult Males From South India

**DOI:** 10.7759/cureus.48283

**Published:** 2023-11-04

**Authors:** Mohamed Javid, Sudhakaran Selvaraj, Ramesh Ganapathy, Senthilkumar Sivalingam, Srikala Prasad, Ananda Kumar Ilangovan, Prabhu Elumalai

**Affiliations:** 1 Urology, Chengalpattu Medical College, Chengalpattu, IND

**Keywords:** voided volume, nomogram, flow rate, uroflow, uroflowmetry

## Abstract

Introduction

Uroflowmetry is a widely accepted non-invasive diagnostic modality used in the evaluation of lower urinary tract dysfunction. While various nomograms have been established for different populations and races, there has been a lack of studies that focus on the South Indian populations. Consequently, the objective of the study was to investigate the urinary flow parameters in a healthy adult male South Indian population using uroflowmetry and identify the variations in flow rates. Additionally, the study sought to assess the influence of age and voided volume (VV) on flow rates and create a representative nomogram.

Methods

A total of 500 uroflowmetry tests were prospectively performed on healthy adult males. The gravimetric method was utilized for uroflowmetry. Flow charts and parameters were analyzed for correlation and linear regression models, and statistical calculations were employed to generate uroflow nomograms.

Results

The mean age of the participants was 37.77±9.91 years. The mean values for maximum flow rate (Qmax), average flow rate (Qavg), and VV were 23.42±6.64 mL/s, 11.71±3.77 mL/s, and 229.90±59 mL, respectively. A significant correlation was observed between flow rates (Qmax and Qavg) and VV, which indicated that increased VV leads to higher flow rates. Additionally, a significant negative correlation between the flow rates and age was noted.

Conclusion

The findings provide insight into the uroflow parameters of the South Indian adult male population and contribute to the development of nomograms, establishing normal reference ranges for flow rates across varying VVs. As a tribute to the hospital and the study participants, the nomogram was named the "Chengai Nomogram."

## Introduction

Uroflowmetry is a non-invasive diagnostic investigation for the assessment of urinary flow quantitatively and the identification of lower urinary tract dysfunction [1]. It serves as a crucial tool for both initial and follow-up evaluations of lower urinary tract symptoms (LUTS) due to its simplicity in utilization and measurement along with its vivid clinical inferences [1-3]. Its easy repeatability makes it suitable for monitoring treatment outcomes [1]. However, the clinical utility of uroflowmetry may be relatively constrained because of the absence of uniformly accepted benchmarks that could be used to define the rates that could be considered normal [3].

Among the various parameters measured by uroflowmetry, the peak flow rate (Qmax), the average flow rate (Qavg), and the voided volume (VV) hold high clinical significance [1]. The urinary flow rate has a notable relationship with the VV of urine, on which it is also dependent [3-5]. This has impelled the development of nomograms to understand the changes in the flow rates based on the VVs [3]. As a result, these nomograms can aid in mitigating the issue of correlating flow rates to only one VV of urine. Factoring in the physiological variations of the bladder and urethra that exist in various populations and ethnicities, the variations in flow rates have been studied and numerous nomograms have also been constructed [1, 3-12]. However, there is sparse data in this regard while focusing on the South Indian population.

Consequently, our study was designed to investigate the urinary flow characteristics in healthy adult male South Indian population utilizing uroflowmetry and analyze the variations in flow rates. Additionally, our study sought to comprehend the normal reference ranges for the Qmax and Qavg, analyze the impact of age and VVs on flow rates, and formulate a corresponding nomogram.

## Materials and methods

Study design and participants

A cross-sectional analytical study was conducted from June 2022 to May 2023 and 500 adult (>18 years of age) male participants who accompanied patients to the urology department for various reasons were prospectively enrolled. The participants who were included did not experience any LUTS and had no previous history of urological diseases, medications, or interventions. We excluded individuals with a background of neurological or psychological conditions; diabetes mellitus; or any specific urological disorders such as LUTS, urinary tract infections, bladder and urethral stones, and meatal stenosis that could potentially alter the structure and function of the normal urinary tract. Additionally, those who underwent previous urological interventions or were on medications that could potentially modify the lower urinary tract function, such as anticholinergics, alpha-blockers, 5-alpha reductase inhibitors, and antibiotics, were also excluded. The study was conducted after the approval from the Institutional Ethics Committee (Ref. No: CMCH-22-PR-376).

Data collection

A total of 500 uroflowmetry tests were systematically performed, and prior to the test, the participants were instructed to have a comfortably full bladder before voiding into the designated uroflowmeter. Adequate privacy was provided during the procedure to negate the development of any psychological influences that might influence the results. Any void from the participant that was suggestive of an intermittent flow or those with a VV less than 50 mL were excluded, as these voids might not be representative of the individual's natural voiding pattern or bladder habit. In such scenarios, the participants were requested for a follow-up session and were asked to repeat the void. Pre-void urine and post-void urine were also measured by ultrasound to ensure that the participants did not have any obvious pathology.

Uroflowmetry

For our study, we utilized the Digital Urodynamic Machine (Solar Silver, MMS International, Netherlands). To ensure and maintain accuracy, regular calibration sessions were undertaken periodically. The main parameters that were assessed during these sessions included the VV, the Qmax, and the Qavg.

Statistical analysis

The collected data were entered into Microsoft Excel (Microsoft Corporation, Redmond, USA) and subsequently analyzed using SPSS statistical software (IBM, Inc., Chicago, IL, USA) Version 27 and R statistical software v4.0.0. Descriptive statistics were utilized where continuous variables were represented as median or mean with standard deviation (SD) in cases of normal distribution, and categorical variables were presented in percentage format. The Pearson correlation coefficient was applied to study the relationships between age, Qmax, Qavg, and VV. For comparisons involving more than two groups, especially age against Qmax and Qavg, one-way ANOVA was used. To predict the characteristics of dependent variables based on multiple independent variables, multiple linear regression was performed.

## Results

Table 1 illustrates the age distribution of the study population. The age of the participants in our study ranged from 19 to 72 years.

**Table 1:** Age distribution of the participants

Age (in years)	Frequency	Percent
18-25	52	10.4
26-35	174	34.8
36-45	170	34
46-55	83	16.6
>55	21	4.2
Total	500	100

Table 2 demonstrates the details of the Age, Qmax, VV, and Qavg in the study population.

**Table 2:** Parameters in the study group Qmax, maximum flow rate (in mL/second); Qavg, average flow rate (in mL/second)

Parameter	Mean±SD
Age (in years)	37.70±10.25
Voided Volume (mL)	226.88±49.35
Qmax (mL/s)	23.49±9.87
Qavg (mL/s)	11.47±3.55

Table 3 demonstrates the correlation between the different study parameters. Age can be seen to be significantly and inversely correlated with VV, Qmax, and Qavg. Whereas VV is significantly and directly correlated with Qmax and Qavg.

**Table 3:** Correlation between age, voided volume, Qmax, and Qavg Qmax, maximum flow rate; Qavg, average flow rate

Pearson Correlation		Qmax	Voided Volume	Qavg
Age	r	-0.098	-0.140	-0.101
	p	0.028	0.002	0.025
Voided Volume	r	0.172		
	p	0.001		
Qavg	r	0.442	0.174	
	p	0.001	0.001	

In multiple linear regression, age was not significant with Qmax (p=0.081) or Qavg (p=0.091), whereas VV was significant with Qmax (p<0.0001) and Qavg (p<0.0001). The regression equations for Qmax and Qavg are Qmax=18.895+0.032VV and Qavg=9.821+0.012VV, respectively.

## Discussion

The development of nomograms is to establish normal reference values for the flow rates at different VVs. In this context, one of the earliest introductions of a nomogram was constructed with the urine flow rates relative to the VV by Siroky et al. [4]. Differences observed between various races and ethnicities have been reported to influence various physiological and pathological processes [13-15]. Furthermore, several personal demographic factors such as height, weight, socioeconomic status, and diet habits have been shown to contribute to the observed differences between various races and ethnicities [13]. As uroflowmetry is one of the fundamental investigations that is used in the evaluation of voiding dysfunction, a precise inference is of paramount importance [1,3]. It is significant to note that nomograms constructed with the data from the regional population can give better insights and a more accurate representation of the urine flow characteristics for that particular region. Our study was prompted by this consideration and to the best of our knowledge, no similar study or data has been documented from the southern part of India.

Of the total study population, the maximum age for participation was within the age group (in years) of 26-35 and 36-45, accounting for 174 participants (34.8%) and 170 participants (34%), respectively. Kumar et al. studied men aged 16 to 50 years, and they reported a mean Qmax of 22.8±9.33 mL/s and a mean Qavg of 13.05±6.12 mL/s [3]. Similarly, Thakur et al. study focused on men aged 15 to 40 years, and they observed a mean Qmax of 24.32±3.50 mL/s and a mean Qavg of 9.45±2.55 mL/s [1]. Our study involved a wider age group of men from 19 to 72 years, and we found a mean Qmax of 23.14±6.07 mL/s and a mean Qavg of 11.47±3.55 mL/s. Despite the variations in age demographics, the parameters observed in our study fall within the range of values reported by Thakur et al. and Kumar et al., without any gross deviation.

Pearson correlation analysis was utilized to study the relationships between age, VV, Qmax, and Qavg. Our findings provided significant insights into the dynamics of the urine flow characteristics in our regional population. It was observed that age had a significant and inverse correlation with Qmax (r=-0.098, p=0.028) and Qavg (r=-0.101, p=0.025). This suggested that as individuals grew older, their Qmax and Qavg decreased. On the contrary, VV exhibited a significant and positive correlation with both Qmax (r=0.172, p=0.001) and Qavg (r=0.174, p=0.001). This indicated that as VV increased, there was a parallel increase in both maximum and average flow rates.

Our study has certain limitations that are worth noting. First, the VVs ranged from 146 mL to 414 mL. Though this might be informative between these values, it may not capture the urinary flow characteristics that lie outside of this range. Second, our study exclusively enrolled only male participants due to regional cultural hesitation from the female counterpart. This potentially limits the generalizability of our findings to the entire population. So we suggest that future research should explore a wider VV spectrum and include both genders to provide a more holistic comprehension of the urinary flow patterns.

**Figure 1:**
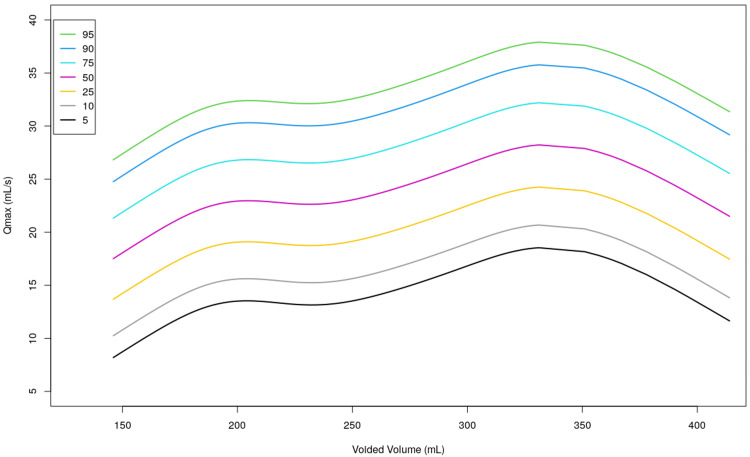
Uroflowmetry nomogram for maximum flow rate in the adult male population of South India The colored lines represent specific percentiles as detailed in the key box. Qmax, maximum flow rate

**Figure 2:**
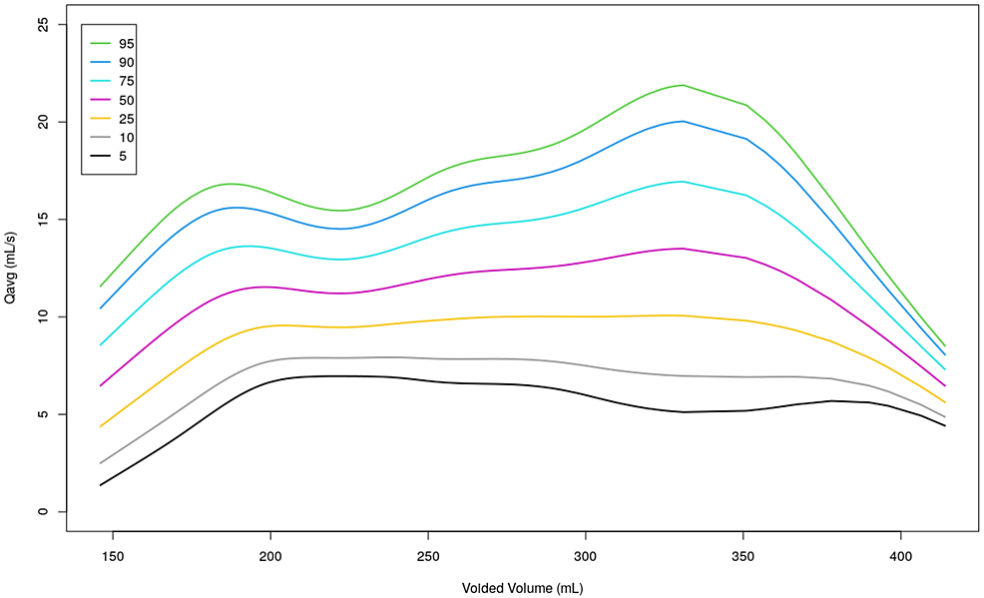
Uroflowmetry nomogram for average flow rate in the adult male population of South India The colored lines represent specific percentiles as detailed in the key box. Qmax, maximum flow rate

A nomogram was constructed using the data that was collected from our study (Figure 1 and Figure 2). In honor of our hospital and the participants who contributed to our study, we suggest identifying the nomogram as "Chengai Nomogram," which is based on the location of our hospital.

## Conclusions

Our study highlights valuable insights into the urinary flow parameters among healthy males in the southern region of India and can serve as a valuable resource for clinicians aiding them in more precise diagnoses and treatment options based on the regional data. Flow rates (Qmax and Qavg) exhibited a significant positive correlation with VV and a significant negative correlation with age. A new nomogram called “Chengai Nomogram” has been created with the region-specific data. 
